# Association between serum markers of the humoral immune system and inflammation in the Swedish AMORIS study

**DOI:** 10.1186/s12865-021-00448-2

**Published:** 2021-09-06

**Authors:** Aida Santaolalla, Sam Sollie, Ali Rislan, Debra H. Josephs, Niklas Hammar, Goran Walldius, Hans Garmo, Sophia N. Karagiannis, Mieke Van Hemelrijck

**Affiliations:** 1 Translational Oncology and Urology Research (TOUR), School of Cancer and Pharmaceutical Sciences, King’s College London, Guy’s Hospital, 3rd Floor, Bermondsey Wing, London, SE1 9RT UK; 2 Department of Medical Oncology, Guy’s and St Thomas’ NHS Trust, London, UK; 3 Unit of Epidemiology, Institute of Environmental Medicine, Karolinska Institutet, Stockholm, Sweden; 4 Unit of Cardiovascular Epidemiology, Institute of Environmental Medicine, Karolinska Institutet, Stockholm, Sweden; 5 Regional Cancer Center, Uppsala/Örebro, Uppsala University Hospital, Uppsala, Sweden; 6 Department of Surgical Sciences, Uppsala University, Uppsala, Sweden; 7 St John’s Institute of Dermatology, School of Basic and Medical Biosciences, King’s College London, Guy’s Hospital, London, UK; 8 Breast Cancer Now Research Unit, School of Cancer and Pharmaceutical Sciences, King’s College London, London, UK

**Keywords:** Immune system, Inflammation, Biomarkers, Interaction, AMORIS, Multivariable analysis, Socio economic factors

## Abstract

**Background:**

Although the onset of inflammatory cascades may profoundly influence the nature of antibody responses, the interplay between inflammatory and humoral (antibody) immune markers remains unclear. Thus, we explored the reciprocity between the humoral immune system and inflammation and assessed how external socio-demographic factors may influence these interactions. From the AMORIS cohort, 5513 individuals were identified with baseline measurements of serum humoral immune [immunoglobulin G, A & M (IgG, IgA, IgM)] and inflammation (C-reactive protein (CRP), albumin, haptoglobin, white blood cells (WBC), iron and total iron-binding capacity) markers measured on the same day. Correlation analysis, principal component analysis and hierarchical clustering were used to evaluate biomarkers correlation, variation and associations. Multivariate analysis of variance was used to assess associations between biomarkers and educational level, socio-economic status, sex and age.

**Results:**

Frequently used serum markers for inflammation, CRP, haptoglobin and white blood cells, correlated together. Hierarchical clustering and principal component analysis confirmed the interaction between these main biological responses, showing an acute response component (CRP, Haptoglobin, WBC, IgM) and adaptive response component (Albumin, Iron, TIBC, IgA, IgG). A socioeconomic gradient associated with worse health outcomes was observed, specifically low educational level, older age and male sex were associated with serum levels that indicated infection and inflammation.

**Conclusions:**

These findings indicate that serum markers of the humoral immune system and inflammation closely interact in response to infection or inflammation. Clustering analysis presented two main immune response components: an acute and an adaptive response, comprising markers of both biological pathways. Future studies should shift from single internal marker assessment to multiple humoral and inflammation serum markers combined, when assessing risk of clinical outcomes such as cancer.

**Supplementary Information:**

The online version contains supplementary material available at 10.1186/s12865-021-00448-2.

## Background

The immune system is a complex set of physiological mechanisms that defend the body against infectious agents (bacteria, viruses, parasites, fungi) that can cause disease. Immunological surveillance can also detect and eliminate cancer cells [1]. Micro-organisms that penetrate the body for the first time are met immediately by cells and molecules that can mount an innate immune response. Macrophages conduct the defence against bacteria and fungi, but also by acting as antigen-presenting cells to trigger adaptive immunity and as effector cells in antibody-dependent cellular cytotoxicity (ADCC) and phagocytosis (ADCP). Cytokines and chemokines released by macrophages in response to pathogen constituents initiate the process known as inflammation [2]. Inflammation is the body’s protective response to injuries and infections [3]. Immature circulating IgM antibodies can also act as a first line of defence. The adaptive immune system consists of cell mediated and humoral (antibody) immunity and can respond to protect from pathogens. Activation of humoral immunity can manifest in the form of serum antibodies, of different classes, including IgM, IgA, IgG and IgE, produced by plasma B cells. Mature class-switched and affinity-matured antibodies can recognise antigens with high affinity, engage immune effector cells to mediate pathogen ADCC and ADCP, and trigger the complement cascade leading to effective elimination of infection [4]. Cell mediated immunity relies on the direct cytotoxic activity of specific immune cells, such as cytotoxic T cells, in conjunction with various immune cells and components [2].

Most immune-epidemiological studies have been geared towards developing an understanding of the consequence of single broad exposures of inflammation in relation to development of specific diseases, defining an exposure as any factor that can be associated with a disease of interest. When considering chronic inflammation, these studies have now been found to be inadequate as there is no single exposure relating to disease aetiology and progression [5]. A more realistic model assessing multiple exposures must be implemented. As such, the concept of the exposome was introduced in 2005 [6], a complete cumulative measure of environmental exposures (both internal and external) and the biological responses they may elicit over an entire life span [7].

Under the exposome framework, epidemiological studies exploring the potential interplay between the humoral immune system and chronic inflammation may be valuable to comprehend their complex relationship, given that different levels of immunoglobulins and specific inflammatory markers are known to be signs of diseases such as autoimmune diseases, diabetes and heart disease. For instance, heavy drinkers with advanced liver disease often present with high IgA values and increases in serum IgA levels are a generalized phenomenon in diabetic patients [8–10]. Smoking and alcohol intake could also affect serum IgA, IgG or IgM. Since serum immunoglobulins are widely reported to be dysregulated in various inflammatory diseases, it is logical for associations of IG levels and inflammation markers to be studied [11–13]. We therefore performed a study to assess how serum biomarkers of the humoral immune system (IgA, IgG and IgM) and serum biomarkers of inflammation (C-reactive protein, albumin, haptoglobin, leukocytes, iron, and total iron-binding capacity) may interplay and how general and external environmental factors such as age, sex, educational level and socio-economic status influence these specific internal factors. All the factors assessed were available for the full cohort and were taken simultaneously in the same day. Other exposures of the immune system and inflammation such as IgE, a well-established marker of allergy, were not measured in most of the participants in the cohort. Likewise, other potential confounders such as BMI, smoking and alcohol consumption were not available to be included in the study.

To our knowledge, this is the first study investigating such associations between serum markers of the humoral immune system and serum markers of inflammation in a healthy cohort.

## Results

Characteristics of study participants are shown in Table 1. The study population consisted of 5513 participants whom had all serum markers of the humoral immune system and inflammation measured on the same day. The study population contained more women (63.30%) than men (36.70%) and the mean age of the participants was 51.4 years. Most of the participants had attained ‘Middle’ (39.96%), compared to ‘Low’ (27.30%) and to ‘High’ (23.67%) level of education. Moreover, 83.98% of the individuals had no diseases recorded at the time the measurements were taken, which implies the healthy status of the study population.

**Table 1:** Descriptive statistics of study population

	N = 5513 (100%)
Sex	
Male	2023 (36.70)
Female	3490 (63.30)
Age	
Mean (SD)	51.4 (17.27)
< 40	1523 (27.63)
40–50	1165 (21.13)
50–65	1460 (26.48)
> 65	1365 (24.76)
SES	
Unclassified/missing	1224 (22.20)
Low	2213 (40.14)
High	2076 (37.66)
Education	
Missing	500 (9.07)
Low	1505 (27.30)
Middle	2203 (39.96)
High	1305 (23.67)
Charlson comorbidity index	
0	4630 (83.98)
1	440 (7.98)
2	293 (5.31)
3+	150 (2.72)

Biomarkers	Mean (SD)
IgA (g/L)	2.65 (1.39)
IgG (g/L)	12.65 (3.91)
IgM (g/L)	1.34 (1.03)
CRP (mg/L)	6.04 (12.63)
Haptoglobin (g/L)	1.14 (0.36)
Albumin (g/L)	42.40 (3.23)
White blood cells (10^9^ cells/L)	6.82 (3.37)
Iron (µmol/L)	16.27 (5.54)
Total iron-binding capacity (µmol/L)	60.27 (8.63)

Participants had both serum markers of the humoral immune system and inflammation measured at the same measurement

*SES* socioeconomic status

Table 2 shows a 9 by 9 scatter plot matrix in which all biomarkers are plotted against each other. All cells with the text in bold indicated correlations above or equal to ± 0.25 that imply interaction and were statistically significant at *p* value < 0.0006 corrected for multiple testing. The strongest positive correlation was seen for CRP and haptoglobin (rs = 0.50). The rest of the correlation were low as follow: haptoglobin and WBC (rs = 0.25) and inverse correlations [haptoglobin and Albumin (rs = − 0.31), CRP and Albumin (rs = − 0.29), CRP and Iron (rs = − 0.28)]. Markers of the humoral immune system, IgG and IgA, showed a low correlation (rs = 0.22). Moreover, low inverse correlations were observed between Albumin and IgA (rs = − 0.26) and IgG (rs = − 0.20).

**Table 2:** Spearman’s rs rank-order correlation matrix between all nine serum markers

	Biomarker correlations								
	IgA	IgG	IgM	CRP	Albumin	Haptoglobin	WBC	Iron	TIBC
IgA	1.00	0.22 *P* < 0.0001	− 0.07 *P* < 0.0001	0.11 *P* < 0.0001	− **0.26** ***P* < 0.0001**	0.16 *P* < 0.0001	0.05 *P* < 0.0001	− 0.01 *P* = 0.4639	− 0.11 *P* < 0.0001
IgG	0.22 *P* < 0.0001	1.00	0.13 *P* < 0.0001	0.07 *P* < 0.0001	− 0.20 *P* < 0.0001	0.02 *P* = 0.1536	− 0.03 *P* = 0.0184	− 0.01 *P* = 0.5675	− 0.07 *P* < 0.0001
IgM	− 0.07 *P* < 0.0001	0.13 *P* < 0.0001	1.00	0.02 *P* = 0.0972	− 0.06 *P* < 0.0001	− 0.04 *P* = 0.0034	− 0.01 *P* = 0.2745	− 0.01 *P* = 0.2809	− 0.01 *P* = 0.4373
CR*P*	0.11 *P* < 0.0001	0.07 *P* < 0.0001	0.02 *P* = 0.0972	1.00	− **0.29** ***P* < 0.0001**	**0.50** ***P* < 0.0001**	0.17 *P* < 0.0001	− **0.28** ***P* < 0.0001**	− 0.11 *P* < 0.0001
Albumin	− **0.26** ***P* < 0.0001**	− 0.20 *P* < 0.0001	− 0.06 *P* < 0.0001	− **0.29** ***P* < 0.0001**	1.00	− **0.31** ***P* < 0.0001**	− 0.08 *P* < 0.0001	0.14 *P* < 0.0001	0.19 *P* < 0.0001
Haptoglobin	0.16 *P* < 0.0001	0.02 *P* = 0.1536	− 0.04 *P* = 0.0034	**0.50** ***P* < 0.0001**	− **0.31** ***P* < 0.0001**	1.00	**0.25** ***P* < 0.0001**	− **0.25** ***P* < 0.0001**	− 0.01 *P* = 0.2710
WBC	0.05 *P* < 0.0001	− 0.03 *P* = 0.0184	− 0.01 *P* 0.2745	0.17 *P* < 0.0001	− 0.08 *P* < 0.0001	**0.25** ***P* < 0.0001**	1.00	− 0.09 *P* < 0.0001	0.02 *P* = 0.0717
Iron	− 0.01 *P* = 0.4639	− 0.01 *P* = 0.5675	− 0.01 *P* = 0.2809	− **0.28** ***P* < 0.0001**	0.14 *P* < 0.0001	− **0.25** ***P* < 0.0001**	− 0.09 *P* < 0.0001	1.00	− 0.06 *P* < 0.0001
TIBC	− 0.11 *P* < 0.0001	− 0.07 *P* < 0.0001	0.01 *P* = 0.4373	− 0.11 *P* < 0.0001	0.19 *P* < 0.0001	− 0.01 *P* = 0.2710	0.02 *PP* = 0.0717	− 0.06 *P* < 0.0001	1.00

Correlations above or equal to ± 0.25 are in bold. All these correlations are statistically significant (*P* value < 0.0006)

*WBC* white blood cells, *TIBC* total iron-binding capacity

**Fig. 1:**
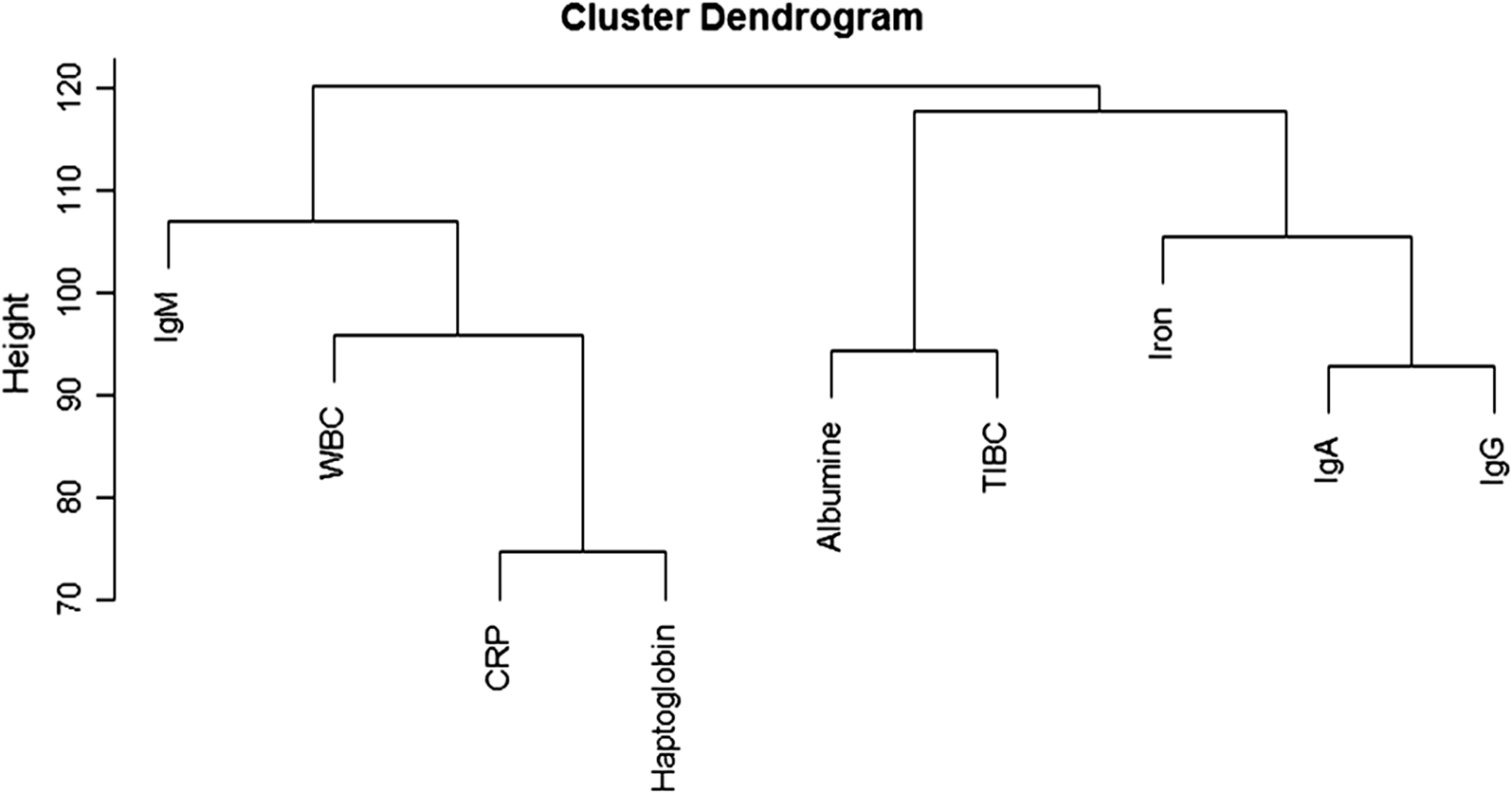
Hierarchical clustering—dendrogram displaying results of hierarchical clustering of all nine serum biomarkers. (*WBC* white blood cells, *TIBC* total iron-binding capacity)

The hierarchical clustering dendrogram is displayed in Fig. 1. Hierarchical clustering formed two large clusters. The first cluster consists of CRP and haptoglobin on the first level. This first level clustered together with WBC on the second level and IgM on the third level. The second cluster consists of two clusters, IgA and IgG, which were clustered together on the second level with iron and albumin and TIBC clustering together.

**Fig. 2:**
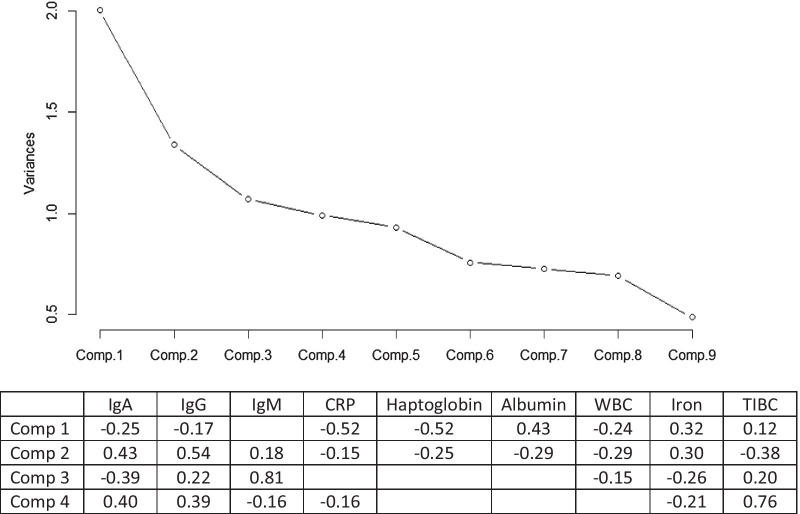
Principal component analysis (PCA)—a scree plot showing the variance of the nine components. The table below is describing the loadings for each of the first four components of the principal component analysis which contain most of the variance of the data. The loadings of each component comprise the weight of each biomarker in the equation that define the particular component. For example, the first component comprises negative weights for IgA, IgG, CRP, Haptoglobin and WBC, and positive weights for albumin, iron and TIBC. (*WBC* white blood cells, *TIBC* total iron-binding capacity)

The scree plot of the principal component analysis displayed the variances of the nine principal components with associated proportion of variance and cumulative proportions (Fig. 2). The first four components produced a cumulative proportion of variance of 61% of the data. The first component had a variance of 2 and accounted for 24% of the entire dataset. Component 2 had a variance of 1.4; 15% of the dataset. Component 3 had a variance of 1.1; 12% of the dataset and component 4 had a variance of 1.0, 11% of the dataset. As the first four components comprised the majority of the variance, no further analysis was performed. The loadings of the first four components presented a first component loaded with the acute inflammatory markers CRP and haptoglobin together with albumin, however presenting an inverse correlation. IgA, IgG and WBC were also contributing with an inverse correlation to this component. The second component was driven by the immunoglobulins, mainly IgG and a smaller inverse contribution of the acute inflammation markers. The third component was loaded mainly with IgM and TIBC (Fig. 2).

The results of the multivariate analysis of variance are displayed in Table 3. Statistical difference in mean was observed for all variables between males and females, except for haptoglobin. The mean values of IgM and iron were not statistically different for the education categories. The mean values of the other biomarkers were statistically different between the low category and the high education category. The mean values of IgG, IgA, albumin and iron markers were statistically different between medium and low education categories, meanwhile the mean values of CRP, haptoglobin, WBC and TIBC markers were statistically different between medium and high education. The mean values of haptoglobin and WBC were not statistically different from each other in the classes of socio-economic status (SES) and the mean values of IgG, Albumin and iron were not statistically different between the low and high category of SES. The mean values of IgA, IgM, CRP and TIBC were statistically different between the low and high category of SES. The mean values for all the serum biomarkers in the different age categories were statistically different from each other. However, the mean values of IgA, IgM, CRP, iron and TIBC were not statistically different from each other between the < 40 and 40–50 age categories.

**Table 3:**
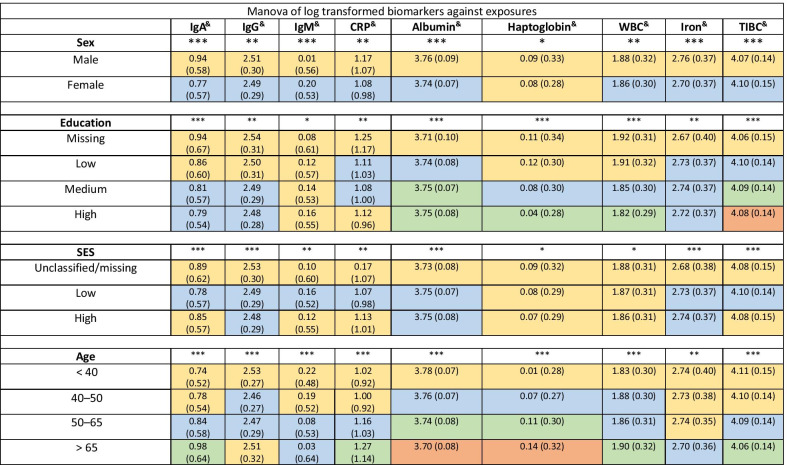
Multivariate analysis of variance (MANOVA)—mean values (standard deviation) in each log transformed biomarker for the given factors sex, education, SES and age

Same colour of cells indicates no statistical difference between categories, meaning that cells that share the same colour, independently of the colour, do not present statistical difference between the variance of the values. On the contrary, cells that present different colour are statistically different (Tuckey’s range test). ***P < 0.0001; **P < 0.05; *P < 0.05

*SES* socio-economic status, *WBC* white blood cells, *TIBC* total iron-binding capacity

^&^All the biomarkers have been log transformed

The interaction analysis presented a statistically significant interaction effect between sex and age in the following markers, IgM, Albumin, WBC and TIBC (Table 4, Additional file 1: Figures S1–S4 and Table S1).

**Table 4:** Manova analysis accounting for interaction between external variables

	IgA	IgG	IgM	CRP	Haptoglobin	Albumin	WBC	Iron	TIBC
SES * education	.1546	.1685	.5025	.4146	.3820	.6234	.2758	.5994	.8433
SES * sex	.0379	.2349	.0524	.8614	.4734	.0075	.9991	.9601	.6564
Education * sex	.5033	.1960	.2852	.6522	.3708	.6628	.0077	.4724	*.0012*
SES * age	.1753	.7472	.7440	.5285	.2541	*.0003*	.0514	.1450	.8552
Education * age	.7581	.0185	.6127	.8787	.2086	.5353	.9512	.6132	.0316
Sex * Age	.1171	.0076	*.0015*	.1113	.2690	< *.0001*	< *.0001*	.6929	*.0002*

*P* value for the manova interaction terms for the independent predictors’ Socioeconomic status (SES), Sex, Age and Education Status. Significant *P* values are highlighted in italics (Bonferroni α/n = .05/15 = .0033)

## Discussion

The study was conducted to understand the need of shifting from single marker approaches into multifactorial analysis when studding the immune and inflammation response in relation to a disease of interest. In order to explore the potential reciprocity between the humoral immune system and inflammation in a healthy population, this study aimed to: (a) evaluate interactions between serum markers of the humoral immune system and serum markers of inflammation; and (b) assess how external socio-demographic factors might influence these biomarkers. Our statistical analyses showed that markers of acute inflammation (CRP, haptoglobin, Albumin and WBC) and markers of the humoral immune system (IgA and IgG) interact and correlate in a complex synergy confirming the close interlink between these biological pathways. Evaluation of the impact of the external environment on the internal environment presented a socio-economic health gradient where lower level of education, older age and male sex were associated with serum biomarker levels that indicated infection and inflammation. This corroborates the essential requirement of deep profiling of the immune and inflammatory response in order to unravel mechanisms leading to complex disease such as cancer. Furthermore, risk stratification tools should consider including large biomarkers panels to be able to inform clinical decision-making [14].

Our findings constitute the first biomarker evaluation that links inflammation and the humoral response.

We report that frequently used serum markers for inflammation, CRP, haptoglobin and white blood cells, clustered together and showed low correlations (rs = ± (0.2–0.5)). CRP and haptoglobin, well-characterised markers known to drive the inflammatory process, correlated most strongly with each other in our analyses. Studies have shown serum haptoglobin and CRP levels to sharply increase in the acute stage of inflammation [15–18]. There is also evidence that serum CRP levels may also be significantly elevated in diseases involving chronic inflammation such as chronic pancreatitis, polycystic ovarian syndrome, obesity and cancer [19–23]. White blood cell count correlated positively with haptoglobin, consistent with evidence that both serum markers have been shown to increase during inflammatory reactions [16, 24]. CRP and haptoglobin correlated negatively with albumin and iron. Indeed, albumin is considered a negative acute phase marker of inflammation [25] and serum iron levels decrease as iron is sequestered during inflammation [26]. Markers of the humoral immune system, IgA and IgG clustered together and presented a low correlation (rs = 0.22). Both immunoglobulin classes generated from B cell maturation and class switching of B cells from IgM/IgD, act as a critical part of the adaptive humoral immune response by specifically recognizing and binding to particular antigens on pathogens and aiding in their destruction [27]. Circulating IgG and IgA are the two most predominant immunoglobulin classes in the human serum. IgG owes its multiple protective properties to its various subclasses which are responsible for fine tuning the nature and strength of the immune response to specific pathogens and respond to antigen challenge in specific anatomical sites. It is plausible that IgG and IgA are regulated at levels dependent on each other during inflammatory responses [28]. Moreover, a negative correlation between IgA and IgG and albumin showed the interaction between these biological pathways. Albumin, a negative acute phase protein, capable of binding ligands, including bacterial products such as lipopolysaccharides, and can possibly modulate the inflammatory reaction in conjunction with IgA and IgG [29].

Hierarchical clustering separated our data in two main groups. The first main group contains serum CRP, haptoglobin, white blood cells and IgM. The results from the correlation analysis are consistent with the formed clusters in this group. CRP and haptoglobin were clustered together and these markers were clustered with white blood cells on the second level. As mentioned previously, these are frequently used serum markers of inflammation. IgM is also in the same cluster. IgM is the first class of antibodies to be produced by B lymphocytes during the innate phase of a humoral immune response. In the absence of somatic hypermutation these IgM antibodies have low affinity, however, their pentameric structure may confer sufficient avidity as the first line of defence against pathogens [28]. All the serum markers in this first main group were biomarkers primarily associated with, or arising from, the primary, acute response in infection and inflammation. Therefore, this first cluster could be linked to non-class switched humoral responses (IgM) and inflammation, indicating less effective immune protection and inflammation that might not effectively progress to class switching and effective humoral response towards immune protection.

The second main group contains IgA, IgG, iron, total iron-binding capacity and albumin. We found a low positive correlation between IgA and IgG. These immunoglobulins were also clustered together in the hierarchical clustering. It is not completely surprising that albumin, iron and TIBC were clustered in the same main group. Albumin levels will be decreased due to inflammation [30]. Serum iron levels will decrease as iron is sequestered during inflammation. As a consequence, the capacity to bind iron will be increased [26]. The serum markers in the second main group were biomarkers primarily associated with, or a result of, an adaptive response to infections and inflammation. This second cluster could explain class switched humoral responses (IgG, IgA), meaning effective mature immunity, with post-acute phase response that might be exemplified in the form of Albumin that may be associated with immune protection.

The principal component analysis indicated that four components contributed only 60% of the variance of the data, so no clear group of variables accounted for large variances in the dataset. However, the first component was loaded with inflammatory markers CRP, haptoglobin and Albumin, the second component with IgG and IgA and the third component mainly with IgM. This indicates the prevalent role of these markers in their specific pathways. The PCA results may be indicative of a significant interplay between the serum markers studied. Serum concentrations may be regulated dependant of or in line with each other.

The statistical analyses were performed to evaluate interactions between serum markers of the humoral immune system and inflammation. The correlation analysis showed that serum markers studied correlated with each other with low rs. Hierarchical clustering supported a transition from acute to chronic inflammation involving interaction between early and late stage markers of inflammation. The principal component analysis supports this notion, as no single biomarker or group of biomarkers contributed the majority of the variance of the data. These findings point to a biological interaction between serum markers of the humoral immune system and inflammation in response to infection or inflammation in the body.

MANOVA indicated that male gender from an older age with a lower level of education were associated with levels of serum biomarkers indicating infection and inflammation, this socio-economic health gradient has been previously described [31]. Males have been shown to have a poorer prognosis than females when it comes to inflammatory disease [32]. Our results show an increase in early markers of inflammation (CRP and white blood cells) compared to females. However, behavioural activity may also play a significant role. Current literature suggests that males are less likely to actively seek out information and medical attention regarding health issues [33]. Participants had higher levels of serum markers of infection and inflammation when they were of the low education group. Medium and high education groups presented with similar levels of serum markers for the marker cluster representing mature or adaptive immune response indicating a lower presence of these responses activated in comparison with the lower education group. These findings are consistent with large epidemiological and public health studies [34–37] suggesting that although there is much public awareness of the implications about smoking, alcohol and exercise, cognitions differ between levels of education [36, 37]. A decline in immune function is directly associated with aging, so older people are more prone to infections and inflammation [38]. The interaction analysis informed of higher values of IgM and albumin in males than in females, however when we considered age, it showed a different pattern for each biomarker (IgM increasing with age and albumin decreasing with age). If we consider those markers representative of the two-axis observed in the hierarchical clustering (acute and humoral responses), it will indicate an increase of the acute inflammatory response and a decrease of the immune responses with age as seen in previous studies [13, 39–41].

The major strength of this study is the large cohort size and its external and internal validity. All participants were from the greater Stockholm area. The serum biomarker analyses were performed in the same laboratory which have used internationally accredited and calibrated methods [42]. All individuals were referred to the laboratory for a health check-up or were outpatients and were generally healthy [43]. The cohort was initiated in 1985 and follow-up continues to present day, allowing multiple events to be recorded throughout this period. Moreover, the AMORIS cohort is representative of the Swedish population and the subset of individuals studied in this project is comparable to the overall AMORIS population [15]. Despite the slightly greater proportion of employed subjects in the AMORIS cohort compared to the general Stockholm population, it can be considered generalizable to other healthy western populations. During the study period, the all-cause mortality was about 14% lower in the AMORIS population than in the general population of Stockholm County when taking age, gender, and calendar year into account. Nevertheless, this healthy cohort effect would not affect the internal validity of our study and any effect is likely to be minor since it has been shown that the AMORIS population is similar to the general working population of Stockholm County in terms of SES and ethnicity [15]. Taking this into account, we can consider that the study population is representative of the Swedish population ensuring the external validity of the analysis.

In addition, this study used a wide range of biomarkers and assessed them as individuals, not placing them in presumptuous groups.

One of the main limitations of this study is that the markers were measured at one point in time. A single measurement will not consider temporal variation. Exposome studies argue that if limited measurements are to be taken then they should be taken during sensitive periods of human development; such as during antenatal development, infant and puberty years [44]. A further limitation is that high-sensitive CRP tests were not available at the time measurements were conducted in the CALAB. CRP levels < 10 mg/L were unquantifiable, which may have resulted in an underestimation of the associations between the serum biomarkers and the external socio-demographic factors. Moreover, other immunoglobulin classes such as IgE and other potential confounders such as BMI, smoking and alcohol intake were not available for most of the participants and could not be included in the study.

## Conclusion

We observed that serum markers related to the acute response of inflammation clustered together. Furthermore, serum markers of the adaptive humoral response to inflammation were correlated and clustered together in two main components: the acute and adaptive immune response. We could not identify a single or group of markers that had a higher weighting over another that could account for the variance of the data. The findings of this study indicate that serum markers of the humoral immune system and inflammation are interlinked in response to infection or inflammation in the body. Future studies should shift from single internal marker assessment to multiple serum markers of the humoral immune system and chronic inflammation combined, when assessing an outcome such as cancer. This would capture a higher percentage of the heterogeneity studied, allow for a better characterisation of the immune response to inflammation triggers, and in theory contribute to better disease prediction, ultimately contributing to better public health strategies.

## Methods

### Study population and data collection

The Swedish Apolipoprotein-related MORtality RISk cohort (AMORIS) includes blood and urine samples from 812,073 subjects. These subjects were residents of Sweden and were predominantly living in the Stockholm county, ranging in age from younger than 20 to older than 80 years old. All laboratory analyses were conducted at the Central Automation Laboratory (CALAB) Stockholm between 1985 and 1996 on a varying number of biomarkers. Participants were either healthy individuals referred for a yearly health check-up by their employers or were outpatients [45]. A more detailed description of the AMORIS cohort is given elsewhere [46–49].

The AMORIS study, a prospective observational study, is a linkage between the CALAB database and 24 different Swedish national health registers, registers of quality of care, and surveys including socio-economic data as well as a questionnaire and biomedical data from number of research cohorts by using the Swedish 10-digit personal identity number [45]. These registers provide baseline data on educational levels (Low, Middle and High), socio-economic status (SES) (Unclassified/Missing, Low and High), comorbidities and emigration. This study complied with the Declaration of Helsinki and was approved by the Ethics Review Board of the Karolinska Institutet.

The inclusion criteria for the current study were defined as follows: all individuals aged 20 or older and with the following measurements (n = 5513): immunoglobulin A (IgA), immunoglobulin G (IgG), immunoglobulin M (IgM), C-reactive protein (CRP), haptoglobin, albumin, white blood cells (WBC), Serum iron (SI) and total iron-binding capacity (TIBC). All measurements were simultaneously taken on the same day. Numerous antigen types are present in the antibody repertoire; in this study we specifically included IgG, IgA and IgM based on their availability in the AMORIS database. It is of interest to note that total IG levels are commonly used in patient monitoring and general health checks to observe the individual health status. To account for other potential diseases that these particular immunoglobulins may not reflect, we also included the Charlson Comorbidity Index in our analyses [50] (see below).

The quantitative determination of IgA, IgG and IgM was done with a turbidimetric determination with reagents (DAKO—Glostrup, Denmark) using a HITACHI 911 automatic analyser (Boehringer—Mannheim, Germany) with a coefficient of variation < 5% (IgA), ≤ 5% (IgG) and ≤ 7% (IgM) [51–53]. Serum CRP and haptoglobin was measured with an immunoturbidimetric assay (reagents from Orion Diagnostics, Espoo, Finland) using fully automated multichannel analyses (for CRP an AutoChemist- PRISMA, New Clinicon, Stockholm, Sweden 1985–1992 and a DAX 96, Technicon Instruments, Corporation, Tarrytown, NY, USA, 1993–1996; for haptoglobin Hitachi-analysers, Boehringer Mannheim, Baden-Wurttemberg, Germany). High sensitivity CRP assays were not available during the period of blood sample collection (1985/1996). Therefore, CRP < 10 mg/L could not be measured precisely. However, the 10 mg/L cut-off has been widely accepted as the upper limit of the health associated reference range. Albumin was measured using the bromocresol green method. Leukocytes were measured by routinely used haematology analysers (STKS Haematology System from Coulter Corporation, Hialeah, FL). Total imprecision calculated by the coefficient of variation was 12% at CRP level 40 mg/L, 5.6% at haptoglobin level 1.1 g/L, < 1.8% for albumin and < 2.7% at leukocytes (WBC) 10 × 10^9^ cells/L [42]. Serum iron was measured via acidification with citric acid in order to dissociate the Fe^3+^ transferrin complex (coefficient of variation < 5%), whereas TIBC (which indicates the maximum amount of iron required to saturate SI transport protein) was assessed by adding Fe^3+^ to the serum. Both markers were assessed with a DAX 96, Technicon Instruments Corporation, Tarrytown, NY, USA, 1993–1996 [54]. From the CALAB database, we also obtained information on age and sex of the participants. Educational level [Low (≤ 9 years), Middle (9–12 years) & High (> 12 years)], socio-economic status (Unclassified/Missing), Low [unskilled + skilled workers (blue collar)] and High [low/intermediate/high non-manual employee (white collar)] and comorbidities coded following the Charlson Comorbidity Index (CCI) based on data from the Swedish National Patient Register were also included in the study. The CCI accounts for 19 diseases where each disease is assigned a certain number of points. The sum of these points gives a score on which different comorbidity levels are created from no comorbidity to severe comorbidity (0, 1, 2, ≥ 3) [19].

### Data analyses

Four main exploratory analyses were conducted to understand the interactions between the different serum markers and also with potential external factors—a method similar to another recently conducted biomarker-based profile study in AMORIS [14]: (1) Correlation coefficients to understand whether there was collinearity between the markers; (2) Hierarchical clustering to assess whether there were homogeneous subgroups in the dataset; (3) Principal component analysis (PCA) to summarise if there was a set with a smaller number of representative variables that collectively explains most of the variability in the original set; (4) Multivariate analysis of variance (MANOVA) to assess the interaction between internal and external markers in the study population.

The age of participants was stratified into four categories for statistical analysis: < 40, 40 < 50, 50 ≤ 65 and > 65. The 50–65 category was used to consider the menopause in females. The > 65 category was added because its clusters retired individuals—a category different to other strata in the AMORIS cohort. This categorisation was considered following previous studies in the AMORIS cohort [54–62]. Comorbidities were assessed using the Charlson Comorbidity Index (mentioned above).

1. Correlations
  Correlations between all serum markers were calculated using Spearman’s rank order correlations to produce a default 9 × 9 data frame matrix between all pairs of serum markers, given that the biomarkers were not normally distributed. Bonferroni correction for multiple testing was applied to estimate the significant correlations.
2. Hierarchical clustering
  To cross-validate the results of the correlations, hierarchical clustering using the Z transformed (mean = 0 and standard deviation = 1) data was carried out using R studio’s defaults package *hclust* for the entire population studied. Hierarchical clustering produced results similar to correlation analysis. Therefore, no further analysis was attempted.
3. Principal component analysis
  Prior to performing principal component analysis, the data was Z transformed (mean = 0 and standard deviation = 1). Principal component analysis was carried out using the *princip* package in R studio. Nine components were produced. A scree-plot graph was produced to display the variances of the nine components. Proportions of variances and cumulative proportions were calculated for the loadings of these components.
4. Multivariate analysis of variance (MANOVA)
  Normality distribution was checked for all serum markers, but as none were normally distributed, all were converted to logarithmic values prior to the multivariate analysis of variance. Statistical differences in mean values for each serum marker and each of the external environmental factors were calculated. Homogeneity of markers was analysed using Levene’s and Welch’s *t* test. Tuckey’s range test was conducted to assess in which categories there was a statistical difference in mean (*p* < 0.05). Moreover, to explore possible interactions between the external factors, a model including the interaction terms was also performed. Furthermore, we assessed the following assumptions: (1) Observations are randomly and independently sampled from the population; (2) Each dependent variable has an interval measurement; (3) Dependent variables are multivariate normally distributed within each group of the independent variables (which are categorical); and (4) The population covariance matrices of each group are equal (this is an extension of homogeneity of variances required for univariate ANOVA). The study met the first two assumptions. The third assumption was explored by testing if the residuals resulted of the MANOVA analysis were normally distributed. The Q–Q plot indicated that the residuals were not normally distributed and hence a log-transformation was used to ensure normal distribution of the data prior the analysis. The fourth assumption was tested using the Levene’s *t* test.
  All statistical analyses were conducted with Statistical Analysis Systems (SAS) release 9.4 (SAS Institute, Cary, NC) and R studio 3.3.2. (R Foundation for Statistical Computing, Vienna, Austria).

## Supplementary Information

**Additional file 1**. Supplementary information describing the interactions among biomarkers.

## Data Availability

Access to data for collaboration is provided by the Steering group members of the AMORIS study by request in email under the heading AMORIS Cohort Collaboration. This can be found at the AMORIS homepage http://amoriscohort.imm.ki.se.

## Abbreviations

AMORIS: Apolipoprotein-related MORtality RISk cohort
CALAB: Central Automation Laboratory
CCI: Charlson Comorbidity Index
CRP: C-reactive protein
IgA: Immunoglobulin A
IgG: Immunoglobulin G
IgM: Immunoglobulin M
MANOVA: Multivariate analysis of variance
PCA: Principal component analysis
SAS: Statistical analysis systems
SES: Socio-economic status
SI: Serum iron
TIBC: Total iron-binding capacity
WBC: White blood cells/leukocytes
