# Supplementary material for: Association between serum markers of the humoral immune system and inflammation in the Swedish AMORIS study
Source: BMC Immunol. 2021 Sep 6;22:61. doi: 10.1186/s12865-021-00448-2 (PMC8420021; doi:10.1186/s12865-021-00448-2)
Supplement: Supplementary file 1 — Additional file 1. Supplementary information describing the interactions among biomarkers. [file 12865_2021_448_MOESM1_ESM.docx]

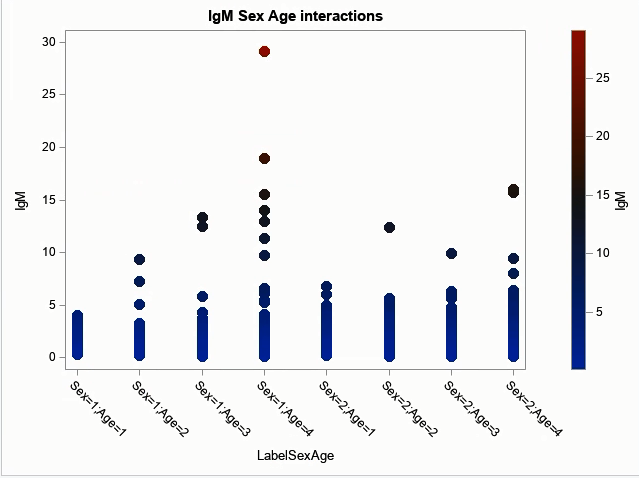


**Figure S1 interactions between Sex and Age for IgM** – Sex and Age interactions were statistically significant in IgM. The plot presents the dependent continuous variable (IgM values Y axis) versus the categories resulted of the combinations of the two independent variables studied (sex and age combinations categories X axis).


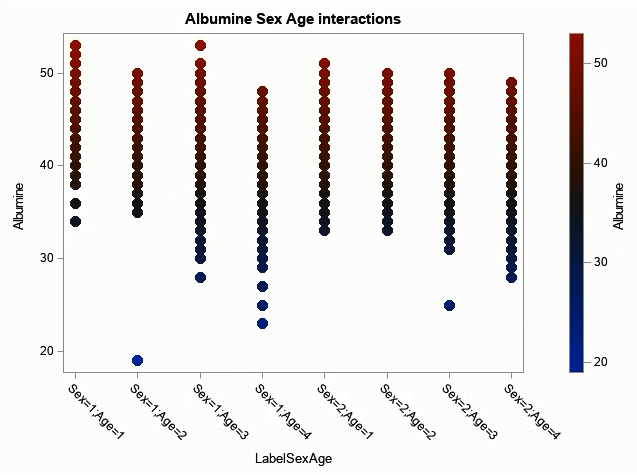


**Figure S2 interactions between Sex and Age for Albumin** – Sex and Age interactions were statistically significant in Albumin. The plot presents the dependent continuous variable (Albumin values Y axis) versus the categories resulted of the combinations of the two independent variables studied (sex and age combinations categories X axis).


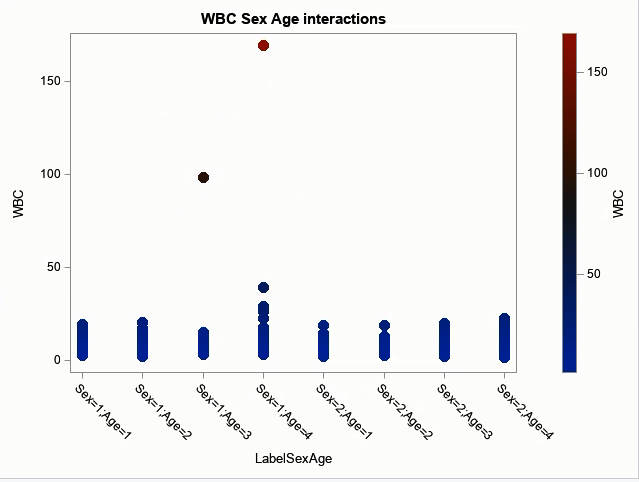


**Figure S3 interactions between Sex and Age for WBC** – Sex and Age interactions were statistically significant in WBC. The plot presents the dependent continuous variable (WBC values Y axis) versus the categories resulted of the combinations of the two independent variables studied (sex and age combinations categories X axis).


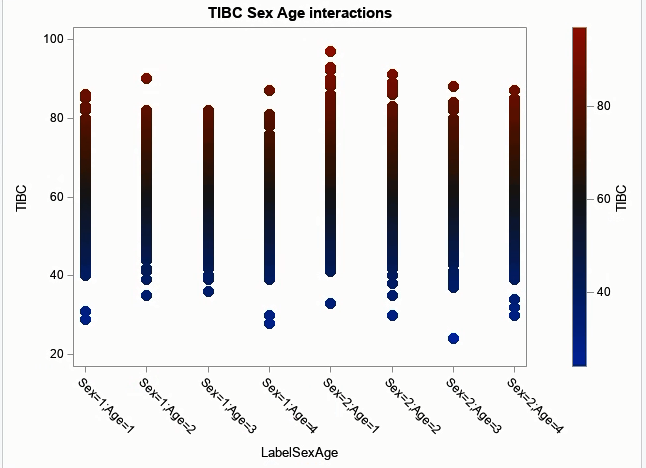


**Figure S4 interactions between Sex and Age for TIBC** – Sex and Age interactions were statistically significant in TIBC. The plot presents the dependent continuous variable (TIBC values Y axis) versus the categories resulted of the combinations of the two independent variables studied (sex and age combinations categories X axis).


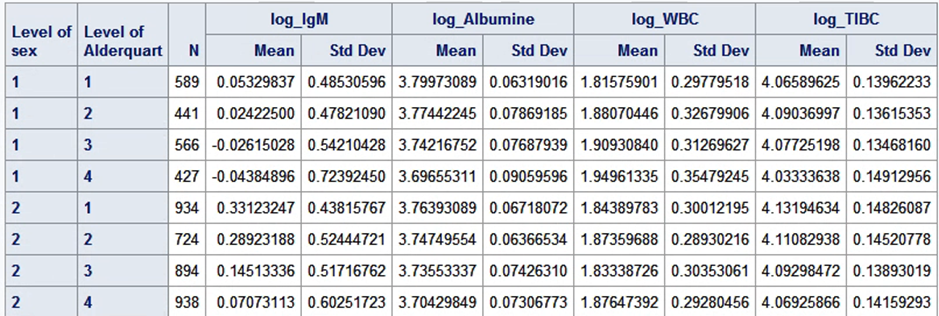


**Table S1 presents the estimates for interaction between Age and Sex for the four biomarker IgM, Albumin, WBC and TIBC**. Please note adelquart is the age variable. We use parametric manova to explore these interactions.
